# Animal Models of Dengue Virus Infection

**DOI:** 10.3390/v4010062

**Published:** 2012-01-09

**Authors:** Simona Zompi, Eva Harris

**Affiliations:** Division of Infectious Diseases and Vaccinology, School of Public Health, University of California, Berkeley, 1 Barker Hall, Berkeley, CA 94720, USA; Email: simona.zompi@gmail.com

**Keywords:** animal models, dengue virus, pathogenesis, immunopathogenesis, antiviral drugs, vaccines

## Abstract

The development of animal models of dengue virus (DENV) infection and disease has been challenging, as epidemic DENV does not naturally infect non-human species. Non-human primates (NHPs) can sustain viral replication in relevant cell types and develop a robust immune response, but they do not develop overt disease. In contrast, certain immunodeficient mouse models infected with mouse-adapted DENV strains show signs of severe disease similar to the ‘vascular-leak’ syndrome seen in severe dengue in humans. Humanized mouse models can sustain DENV replication and show some signs of disease, but further development is needed to validate the immune response. Classically, immunocompetent mice infected with DENV do not manifest disease or else develop paralysis when inoculated intracranially; however, a new model using high doses of DENV has recently been shown to develop hemorrhagic signs after infection. Overall, each model has its advantages and disadvantages and is differentially suited for studies of dengue pathogenesis and immunopathogenesis and/or pre-clinical testing of antiviral drugs and vaccines.

## 1. Introduction

Dengue is the most prevalent arthropod-borne viral illness in humans, with half of the world’s population at risk for infection and up to 50 million cases of dengue estimated each year [1]. Most prevalent in tropical and sub-tropical areas of Latin America and South/Southeast Asia, dengue continues to spread into new areas. Outbreaks occurred in 2009 and 2010 in the Key West islands in Florida [2], and sporadic autochthonous cases were reported in the Mediterranean region, in southern France and Croatia in 2010 [3,4]. Finally, endemic areas have been reported in the Eastern Mediterranean [5,6]. Dengue is caused by four DENV serotypes, DENV-1-4, which circulate concomitantly in different regions of the world. They are transmitted by *Aedes aegypti* and *Ae. albopictus* mosquitoes, the latter of which has become endemic in the Southern part of the US [7], prompting the re-classification of DENV as a Category A pathogen. DENV is a member of the *Flaviviridae* family and is related to yellow fever virus (YFV), hepatitis C virus (HCV), and the West Nile (WNV), Japanese (JEV), and St. Louis encephalitis viruses.

Human infection with DENV results in either asymptomatic or symptomatic disease, ranging from classical dengue fever (DF) to more severe cases of dengue hemorrhagic fever (DHF) and dengue shock syndrome (DSS). DF is a self-limited, though incapacitating, febrile illness accompanied by retro-orbital pain, headache, skin rash and bone and muscle pain. The hallmark of the more severe forms of the disease is a vascular leak syndrome. In the case of DHF, hemorrhagic manifestations, low platelet count, and signs of vascular leak, such as increased hematocrit level or pleural effusion, accompany the symptoms of DF. Evidence of hypotension, signs of shock, and circulatory failure indicate progression to DSS [8]. The WHO has recently revised its classification schema [6], which now consists of Dengue With or Without Warning Signs and Severe Dengue. Overall, this new classification is helpful for clinical management of potentially severe cases [9,10], but the very broad definition of Severe Dengue complicates studies of dengue pathogenesis [10]. 

Development of a suitable animal model for DENV infection has been hampered by the low level or lack of replication of DENV clinical isolates in wild-type (WT) mice and the lack of clinical disease in non-human primates (NHPs). Initial models in mice used intracranial (i.c.) high-dose injections of neurovirulent DENV strains into suckling mice [11] and into adult immunocompetent mice, which induced neurological disease and paralysis [12]. Peripheral replication of DENV in mice was first reported by intravenous (i.v.) and sub-cutaneous (s.c.) infection using a mouse deficient for both IFN‑α/β and -γ receptors in a 129 background (AG129) [13]. Generation of mouse-adapted strains improved this model, allowing study of pathogenesis [14,15,16] and testing of therapeutic antibodies (Abs) and other antiviral compounds [14,17,18,19,20]. The engraftment of human hematopoietic progenitors in immunodeficient mice allowed the development of humanized mice, facilitating infection with clinical DENV isolates and approximating dengue-like illness [21,22,23,24]. Although NHPs do not develop any clinical signs of disease, DENV infects relevant cells, and this model is useful for investigating the immune response to DENV infection and for testing vaccines in pre-clinical trials. We will discuss the advantages and disadvantages of each model and review the applications for which each model has been used thus far.

## 2. Animal Models Used for Dengue Tropism and Pathogenesis Studies

Clinical manifestations of DENV infection and disease severity vary according to different factors: (1) host, including genetic, factors; (2) features related to the virus, including virulence and cellular tropism; (3) immune response, including both protective and enhancing responses. Manipulation of the virus and the host has allowed the development of animal models suitable for pathogenesis studies. 

### 2.1. DENV Tropism and Severe Disease Manifestations in Humans

DENV is delivered intradermally (i.d.) through a mosquito bite and initially infects dendritic cells (DCs) and macrophages. The use of Abs directed against NS3, a non-structural DENV protein, allowed the study of DENV tropism by identifying cells in which active DENV replication was occurring [25]. Macrophages and dendritic cells (DCs) were infected in human autopsy specimens, both in the spleen and lymph nodes [25]. Similarly in mice, NS3 was detected in phagocytes of the spleen and lymph nodes and myeloid cells in bone marrow [25]. These cells migrate to the lymph nodes where DENV replicates before spreading to the rest of the body. Bone marrow myeloid cells and hepatocytes in liver were also found to be infected [25]. These findings are consistent with previous studies of tropism in human autopsies using Abs directed to the DENV E protein [26,27,28,29,30,31,32,33]. These findings are also consistent with observations in NHPs showing that mononuclear phagocytes are the site of viral replication *in vivo* [34]. In a mouse model of Ab‑enhanced DENV infection, DENV replication in sinusoidal endothelial cells in the liver was reported [16], though the relevance to human disease has yet to be established.

Clinical signs of dengue include fever, rash, thrombocytopenia (platelet count ≤ 100,000 cells/mm^3^), elevated liver enzymes, bleeding and plasma leakage. Thrombocytopenia is one of the major signs of DENV infection, and can help with differential diagnosis, especially in young children [35]. Thrombocytopenia can be found across the spectrum of disease, from DF to DSS, but platelet counts lower than 50,000 cells/mm^3^ are considered a sign of severe disease, and severe thrombocytopenia correlates with vascular leak syndrome [36,37]. The mechanism of thrombocytopenia has not yet been elucidated, and several causes have been explored: Platelet-associated IgG [38]; auto-immunity with anti-platelet Abs, eventually resulting in thrombotic microangiopathy [39,40]; and disseminated intra‑vascular coagulation (DIC) [41]. Decrease in platelet count has also been attributed to bone marrow suppression, induced early after infection and before onset of fever, and to hemophagocytosis after defervescence [42].

Hemorrhagic manifestations can be the consequence of either thrombocytopenia or vascular damage. Studies of capillaries in skin biopsies from patients with DHF show marked distortion and swelling, but severely damaged vessels are not observed [43]. *In vitro* infection of endothelial cells [44,45,46] has also been reported, although the *in vivo* significance of these findings is not entirely clear [25]. Thrombomodulin is a marker of endothelial damage, which increases in patients with DIC [47] and in DENV-infected patients [48]. The development of DIC during DENV infection is still controversial. While the amount of fibrinogen is often reported as decreased during DENV infection, prothrombin time (PT) is only moderately increased [37]. In addition, fibrinolysis can be secondary to plasminogen activation, and DENV has been shown to directly activate plasminogen [49]. Additionally, anti-plasminogen Abs have been found to correlate with hemorrhagic manifestations during DENV infection [50,51]. Finally, anti-NS1 Abs, directed against the secreted non-structural NS1 protein, have been shown to cross-react with different coagulation components and cells, such as endothelial cells, platelets and fibrinogen [105,107]. 

The exact cause of vascular leak, which peaks at defervescence and can lead to hypovolemic shock, is unknown. A cytokine storm with increase in TNFα and a dysregulation of the immune system have been shown to participate in the pathogenesis of DENV-induced vascular leak [52]. Activation of complement and complement anaphylatoxins can also induce vasodilation and vascular leak [53]. Glycosaminoglycans, which include heparan sulfate, a receptor for DENV adhesion, are negatively-charged polysaccharides, which constitute a physical and electrostatic barrier against leakage of protein from the vascular wall. Disruption of glycosaminoglycans or neutralization of their negative charge via binding of cationic proteins have been proposed to be involved in the pathogenesis of DENV-induced vascular leak [54]. Finally, activation of platelet-activating factor (PAF), has been shown to induce vascular permeability [55]. 

Overall, while DENV tropism has been defined in both human and animal models, the main site of viral replication is still elusive. In addition, the exact mode of pathogenesis of severe disease remains unclear and several mechanisms appear to be implicated in the development of hemorrhagic manifestations and vascular leak syndrome.

### 2.2. NHP Models of DENV Infection

Studies using WNV have shown that mosquitoes inoculate between 10^4^ and 10^6^ PFU of WNV per bite [56], and similar levels of transmission have been assumed for DENV; thus, infection of animals with 10^4^–10^6^ PFU of DENV is usually considered to mimic the inoculum in a mosquito bite. After subcutaneous (s.c.) inoculation with a dose of 10^5^ PFU of DENV, NHPs can sustain viral replication; however, viral replication is much lower than in humans, is limited to lymphoid-rich tissues, and is accompanied by lymphadenopathy, lymphocytosis and leukopenia [57]. Interestingly, the use of cyclophosphamide, an alkylating agent and immunosuppressive drug that primarily affects proliferating cells and induces lymphopenia, enabled DENV infection of monocytes in Rhesus monkeys for prolonged periods [34]. Some Rhesus macaques occasionally displayed low platelet counts, but no other overt clinical signs after s.c. DENV infection were observed [58,59]. More recently, inoculation with a higher dose of DENV via an intravenous (i.v.) route induced hemorrhage and coagulopathy [60]. Monkeys showed hemorrhagic signs by day 3 post-infection, including petechiae and hematomas, coagulopathy with increased D-dimers related to DIC, but no other signs such as fever, anorexia or lethargy [60]. Thus, a high inoculum of DENV injected i.v. in Rhesus macaques can induce hemorrhagic signs reminiscent of hemorrhagic manifestations seen in humans and could potentially be used for pre-clinical testing of therapeutic interventions specifically targeting DENV-associated coagulopathy.

### 2.3. Mouse Models

The initial mouse models of dengue did not reflect clinical signs of human DENV infection but rather developed neurotropic disease, which is not generally observed in humans [11,12]. WT immunocompetent mice, including A/J, BALB/c and C57BL/6 mice, support some level of DENV replication. A/J and BALB/c mice ultimately succumb from paralysis [61,62]. DENV infection in these mice induced limited DENV-specific pathogenesis such as liver damage, as defined by increased liver enzymes, increased white blood cell (WBC) counts, thrombocytopenia, and an increase in hematocrit level reminiscent of vascular leak [62,63,64]. DHF-like disease has been reported in BALB/c mice with high doses of a highly adapted virus strain [55,61]. Hemorrhagic signs were also observed in C57BL/6 mice after infection with very high doses of DENV (3 × 10^9^ PFU of DENV-2 strain 16681) injected intra-dermally (i.d.) in 4 different injection points [65,66]. In addition, in this model the authors demonstrated DENV infection of endothelial cells [65]. Infection of these immunocompetent mice with high doses (3 × 10^9^ pfu/mL) of DENV can also induce endothelial damage and tissue hemorrhage without overt signs of disease [66].

Severe combined immunodeficiency (SCID) mice lack both humoral and cellular responses due to deficiencies in B and T cell development. These mice can sustain xeno-grafts and have been used to study several other viral infections that otherwise lack a suitable small animal model [42]. SCID mice are engrafted with tumor cells prior to DENV infection, and DENV is inoculated into the engrafted cells. While DENV can replicate efficiently in SCID tumor models, including K562-engrafted SCID mice [67], Huh7-engrafted SCID mice [68] or HepG2-engrafted SCID mice [69], DENV infects mainly the engrafted-tumor cells and virus then spreads to the brain, inducing paralysis. Thus, this model is not relevant for tropism and pathogenesis studies, but can be useful for drug and vaccine testing (see below). 

As DENV infects human cells better than mouse cells, another approach has been the development of humanized mice, *i.e.*, mice engrafted with human progenitor cells, such as CD34^+^ cells. SCID mice can easily be engrafted with tumor cells; however, engraftment of human tissues in these mice is not optimal due to a strong innate immune response, including Natural Killer (NK) cells, which hinders engraftment of hematopoietic tissues. Therefore, SCID mice were backcrossed with non-obese diabetic (NOD) mice, which have defects in NK cell function and a deficiency in hemolytic complement response due to the lack of C5, and subsequently with IL2Rγ-knock-out (KO) mice. The combination of a deficient innate and adaptive immune response allows better engraftment of a variety of human cells and tissues. Reconstitution of NOD/SCID/IL2RγKO mice with CD34^+^ human progenitor cells enables the development of different human cells in the mouse, including DCs, one of the main targets of DENV infection [21,23,24]. DENV infects human cells in different mouse tissues, including spleen, bone marrow and liver, all tissues relevant to DENV pathogenesis. After DENV infection, these mice develop clinical signs of DF including fever, erythema and thrombocytopenia [23,24], although no severe disease has been reported yet [23,24]. The advantage of these mice is the possibility of studying the human immune response *in vivo*. However, so far it has been difficult to generate a sustained and sufficient Ab production [23,24]. Although Ab production is difficult to obtain, these mice usually develop a DENV-specific T cell response [70], making it a useful model to study the role of cross‑reactive T cells in sequential infections. In contrast, RAG-hu mice, which lack both RAG and IL2Rγ genes, developed fever but no other signs of disease using a pool of four different low-passage DENV-2 clinical isolates or one lab-adapted DENV-2. Interestingly, most of these RAG/IL2Rγ ΚΟ mice (10 of 16) developed anti-DENV Abs, and 3 of them demonstrated neutralizing Abs [22].

Infection of AG129 mice by DENV clinical isolates induces neurological disease, as seen in other models [13,71]. However, using a mouse-adapted DENV-2 strain designated D2S10, a vascular leak syndrome was induced in AG129 mice, reminiscent of the severe disease seen in humans [15]. The increased virulence of DENV-2 D2S10 has been attributed to mutations in the heparin sulfate-binding domain of the envelope (E) protein that reduce the rate of viral clearance from the periphery [72]. Subsequently, a triple-plaque-purified clone of DENV-2 D2S10, designated S221, which contains an additional mutation in NS1, was reported to cause morbidity in A129 mice, lacking only the IFN-α/β receptor. Additional passage in mice of DENV-2 D2S10 led to selection of another mutation in the heparan-binding region of E and resulted in a virus that causes lethal disease in 129 or C57/BL6 mice lacking only the IFN-α/β receptor [73]. Another group reported signs of severe disease, including liver damage, hemorrhagic signs, and vascular permeability, in BALB/c mice using a highly mouse-adapted DENV-2 strain [55], although the mutations responsible for this increased virulence have not yet been identified. Recently, non-mouse adapted DENV isolates have been shown to induce severe disease in immunocompromised mice. Infection of AG129 mice intraperitoneally (i.p.) with a variable dose (10^2^ to 10^7^ pfu) of the DENV-2 strain D2Y98P (passage 20) induced dose-dependent symptoms, with the development of either non-severe or severe disease, characterized by increased hepatic enzymes and disrupted splenic architecture, with no neurological disease [74]. A mutation in NS4B was later identified as responsible for the virulence and disease severity induced by this virus [75]. 

## 3. Animal Models Used for Immunopathogenesis Studies of Dengue

Epidemiological studies have demonstrated a clear correlation between severe disease and secondary DENV infection with a distinct DENV serotype [76,77,78,79]. Under certain conditions, cross‑reactive anti-DENV Abs can induce Ab-dependent enhancement (ADE) of infection, resulting in more severe disease [80,81]. ADE increases viral load by enhancing viral infection of Fcγ receptor-bearing cells [14]. Cross-reactive T cells likely contribute to immunopathogenesis of severe disease by participating in a pro-inflammatory cascade, with cross‑reactive T cells producing higher levels of TNFα and IFN-γ than homotypic T cells, leading to dysregulation of cytokine production [52,82,83]. In spite of this situation, most of secondary DENV infections in humans are either asymptomatic or manifest as classic DF. Thus, the adaptive immune response is not only implicated in immunopathogenesis but can also induce effective protection. The factors that distinguish cross‑protective immunity from immunopathogenesis are not clearly understood and remain one of the main questions to be answered in order to understand the pathogenesis of severe disease as well as the mechanisms of protection, which could potentially contribute to better vaccine design.

### 3.1. NHP Models

NHPs do not develop any clinical signs of disease; however, they do develop an Ab immune response similar to the immune response seen in humans [57]. In humans, primary infection with each of the 4 DENV serotypes induces a broadly cross-reactive Ab and T cell response, though the response is directed mainly against the infecting serotype [17,84,85]. Cross-reactivity with multiple serotypes is common, especially at epitopes in the more highly conserved NS proteins or conserved epitopes in E, but can vary among different epitopes and depending on the degree of homology found within each DENV serotype/genotype [17,99,100]. Interestingly, sequential infections in NHPs induced a cross‑reactive response, with the highest Ab titers directed against the primary infecting serotype [86,87], reminiscent of what has been shown in humans and mice and termed “original antigenic sin” [88,89,90,91,92]. Viremia increases after a secondary DENV infection in NHPs, suggesting that ADE may increase viral load through cross-reactive Abs [59]. Similarly, after passive transfer of anti‑DENV monoclonal Ab 1A5 prior to DENV infection, viremia increased 3- to 100-fold in Rhesus macaques, though no signs of disease were apparent [93]. T cell responses have been less well characterized in NHP models. One report demonstrated cross‑reactivity of T cells upon DENV infection, with higher responses directed towards the primary infecting serotype [87], mirroring what has been reported in humans, where hyper-activation of low‑affinity memory T cells induced during the primary DENV infection participate in the immunopathogenesis of DHF and DSS [52,94]. In summary, while increased viremia can be demonstrated in NHP models, the animals do not develop vascular leak or syndromes resembling DHF or DSS, thus limiting their use in the study of pathogenesis/immunopathogenesis.

### 3.2. Mouse Models

AG129 mice, though partially immunocompromised, develop a broadly cross-reactive and long‑lasting Ab response to DENV [95]. Sequential DENV infection in this mouse model results in decreased viral load of the second serotype [95] as well as full protection against lethal infection [91]. In addition, T cells have been shown to play a role in protection, as DENV-1-immune mice depleted of T cells prior to a DENV-2 D2S10 lethal infection developed clinical signs of disease [91]. Thus far, the conditions for modeling severe disease after a sequential DENV infection have not yet been established, but will be a great addition for the study of immunopathogenesis induced by cross-reactive T cells. In contrast, ADE has been achieved in AG129 and other mouse strains by passive transfer of anti-DENV monoclonal Abs, cross-reactive immune serum, or diluted homotypic serum prior to infection [14,16,73]. Mice previously primed with anti-DENV serum develop increased viral load in the serum and several tissues and succumb after infection with a sub-lethal dose of DENV (10^5^ PFU DENV-2 D2S10 i.v.). A vascular leak syndrome, increased serum levels of TNFα and IL-10, and low platelet counts precede death [14], signs that are reminiscent of DHF and DSS. All these signs are also induced in a direct lethal model using a 10^7^ PFU dose of DENV-2 D2S10, suggesting that pathogenesis is triggered by high viral load [15], whether this high viral load has been induced by a high DENV inoculum or has been achieved through ADE. Of note, mice receiving passive transfer of serum or Ab do not have a memory response to DENV. It is possible that inducing ADE in the presence of a memory response may be more restricted, and models of sequential DENV infection may be useful to study ADE in the presence of a cellular memory immune response. Overall, the amount of neutralizing Ab seems to be critical for the induction of protection or ADE [14]. Humoral memory response to DENV in humans has been shown to wane over time [77], and the time elapsed between the primary and secondary infection may be a key component in the development of severe disease [80]. Mouse models are not ideally suited to address this question due to the limited lifespan of a mouse; however, development of NHP models would be useful to study this issue. Other key questions that are under investigation are identification of Ab epitopes that are more likely to induce protection *versus* ADE, as well as which serotype sequences are more associated with increased disease severity. While epitope specificity may vary between humans and mice, we have shown that human Abs can induce ADE in mice [17], paving the way towards analysis of human serum in mice [96]. 

The role of cross-reactive T cells in mouse models is beginning to be explored. Homotypic CD8^+^ T cells have been shown to protect against an infection with the same serotype [97]. In contrast, CD4^+^ T cells can help CD8^+^ T cell responses after vaccination but are not necessary for Ab production and CD8^+^ T cell response after primary infection [98]. Using a tumor-engrafted SCID mouse model, T cells have been shown to have both protective and pathogenic roles, inducing either an accelerated death (80% of infected mice) or full recovery from disease (in 20% of the mice) after transfer of DENV-specific T cells [69]. Similarly to what has been described in humans, sequential infections of BALB/c mice induced a cross-reactive CD8^+^ T cell response, which was higher against the first infecting serotype [99]. The magnitude of this T cell response as well as the immunodominant epitope response varied with the sequence of infecting DENV serotypes. However, direct transposition to humans may be difficult as immunodominant epitopes may be different in mice and humans. The preferential activation of cross-reactive T cells during secondary infection in mice induced higher levels of TNFα responses, suggesting participation of cross-reactive T cells in the immunopathogenesis of vascular leak syndrome [99]. Cross-reactive CD4^+^ T cells have also been shown to increase cross‑reactive CD8^+^ T cell responses in this mouse model, suggesting that they may also participate in the immunopathogenesis of severe disease [100]. As with Ab epitopes, T cell epitopes may play differential roles in protection and enhancement, as shown recently in a C57BL/6 model [101]. While these mouse models have shed light on the activation of cross-reactive T cells during secondary DENV infection, none of the mice developed severe disease; thus, a mouse model that supports both viral replication and natural T cell responses and reproduces features of severe disease is needed to better characterize the role of the T cell response during secondary DENV infection and vascular leak. To this end, we have recently developed a new mouse-adapted strain that induces lethal disease in IFNAR1^−/−^ mice (lacking only the IFN-α/β receptor) in both 129 and C57/BL6 backgrounds, setting the stage for potential T cell studies [73]. Recently, a new model to study DENV-specific human T cell responses in mice has been established by back-crossing HLA transgenic mice into the IFNAR1^−/−^ background. Immunodominant T cell epitopes relevant for human studies have been identified using this model [102]. 

NS1 is a non-structural DENV protein that is secreted from infected cells and found attached to the cell surface during DENV infection. The amount of circulating NS1 has been correlated with more severe disease [103], though whether this is due to a direct effect of NS1 or a reflection of higher viremia is not fully understood. Soluble and membrane-bound NS1 have been shown to induce complement activation, increasing vascular leakage [104]. Anti-NS1 Abs have also been shown to play a pathogenic role [105], as some Abs cross-react with endothelial cells and platelets, potentially inducing both thrombocytopenia and vascular leak [105]. In addition, damage of endothelial cells in the liver induced by anti-NS1 Abs has been shown to correlate with liver damage and increased hepatic enzymes in mice [106]. Anti-E Abs have also been shown to interact with plasminogen, inhibiting plasmin activity [107,108]. AG129 mice infected with DENV have high levels of circulating NS1 and anti-NS1 Abs [109], making it a good model to study the role of NS1 and anti-NS1 Ab *in vivo*.

## 4. Animals Models Used for Therapeutic Testing

No anti-viral drug against DENV has been commercialized so far, and only supportive care, including fluid replacement, is used for the management of DHF/DSS, requiring hospitalization. A number of anti-viral candidates have been tested in mouse models that develop paralysis and encephalitis [110,111,112]; however, the relevance to human disease of drugs that target neurological manifestations is not entirely clear, as bioavailability of the drug may be different in the central nervous system than in other tissues. An aqueous extract of neem leaves inhibited viral replication and prevented clinical signs of disease in suckling mice infected with DENV i.c. [113]. Castanospermine, an alpha-glucosidase inhibitor, inhibits the folding of viral proteins and decreases viral secretion and has been shown to prevent mortality in A/J mice infected by the i.c. route [112]. Subsequently, this same drug, together with other anti-viral compounds, was tested in AG129 mice infected with a non‑adapted DENV strain (TSV01) and showed efficacy by reducing viral load and decreasing the inflammatory response [19]. The non-adapted DENV strain TSV01 is well-suited for measuring the effects of potential anti-virals on viremia and has proven useful in demonstrating synergy among different anti-viral compounds when using drug combinations [18].

The AG129 mouse model infected with mouse-adapted DENV strains, leading to a lethal vascular leak syndrome, has also been used for anti-viral testing [19,48,114,115,116,117]. Morbidity, mortality, viral load, signs of vascular leak, platelet count and levels of pro-inflammatory cytokines, such as TNFα, can all be used as relevant read-outs for drug efficacy. Recently, the use of siRNA targeting the conserved 5' cyclization sequence in the DENV viral RNA genome delayed mortality and substantially reduced viral load in several tissues in the AG129 model using ADE-induced infection [118]. We have shown using this same model that monoclonal Abs unable to bind to Fcγ receptors have prophylactic and therapeutic activity [14,17]. Thus, immunodeficient mouse models that develop disease features resembling human disease are valuable in the development of anti-viral drugs against dengue.

## 5. Animals Models Used for Vaccine Testing

There is no DENV vaccine currently licensed, but numerous vaccines are in development and several are in Phase 1/2 of clinical trials. As the immune mechanisms of protection against DENV are not fully understood, clear correlates of immune protection are not yet available to monitor vaccine efficacy/effectiveness. Neutralizing Abs are important for protection against DENV infection, but the amount and characteristics of Ab needed for full protection remain elusive. In addition, animal models have demonstrated the contribution of cytotoxic T cells to protection, but further investigation is needed in the human system. Because of the risk of developing more severe disease after a secondary infection due to cross-reactive Ab or cross-reactive T cells, a successful dengue vaccine needs to induce a balanced response against the four serotypes of DENV. Finally, waning immunity can induce ADE in the long term as the amount of anti-DENV Abs decrease; thus, induction of long-lasting immunity is an issue that must be addressed. 

DENV vaccines developed thus far are either live-attenuated vaccines, inactivated vaccines, virus‑like particles (VLPs) or subunit vaccines targeting the viral envelope (E), pre-membrane (prM) or NS1 proteins [119,120,121,122]. Pre-clinical vaccine testing includes evaluation of safety, infectivity (for the live-attenuated vaccines), immunogenicity and efficacy. Due to the high costs of NHP models, mouse models are usually used as a first step for pre-clinical development of vaccines. 

Immunocompetent mice are in general the best-suited models to test the immunogenicity of a vaccine and have been proven to be useful for testing subunit vaccines [123,124,125,126,127]. However, DENV replication is low in these mice; thus, the immune response induced by live-attenuated vaccines may be underestimated due to the low level of viral replication. As discussed above, immunocompetent mice, either adult or suckling mice, infected with DENV i.c. develop neurological signs and succumb by paralysis. This end-point has been chosen as a read-out to measure vaccine efficacy in several studies [65,68,128]. In addition, suckling mice are a good model for safety testing, in order to evaluate the potential neurovirulence of live-attenuated dengue vaccines [129]. SCID mice tumor cell models of infection have similarly been used to assess vaccine effectiveness by measuring viral load after challenge [68,130]. This model has also been used to test attenuation of virulence, as measured by the duration and magnitude of the viremia; however, it may be less sensitive than the suckling mouse model, as several candidate vaccine strains that were greatly attenuated in the SCID-tumor model were not attenuated when tested in NHPs [131].

The immunocompromised mouse model AG129 was originally developed for vaccine testing since these mice can sustain DENV replication [13], though as initially tested, they succumbed to paralysis. Generation of a mouse-adapted DENV strain enabled development of a mouse model that manifests signs of severe dengue reminiscent of human disease, including vascular leak [15]. Thus, this model of systemic dengue disease may constitute a more satisfactory approach for testing vaccine efficacy. However, concerns have been raised regarding the development of a full immune response due to the lack of IFN-α/β and -γ receptors. Nevertheless, we have shown that AG129 mice develop DENV‑specific Abs that are long-lasting and protective against homologous and heterologous viral challenge [95]. In addition, we have shown that the IgG isotype composition of the Abs produced by AG129 is fairly balanced (IgG1:IgG2a:IgG2b = 1:4:1) and similar to what has been reported in WT mice [132]. AG129 mice also develop a strong B cell response, as measured by ELISPOT [91]. The T cell response has not been extensively characterized in these mice, as it is known that IFN-γ is necessary for correct recall of the memory T cell response [133]. Despite this, we have shown a certain degree of T cell functionality, as depletion of T cells prior to DENV infection induced morbidity, while non-depleted mice were fully protected and showed no signs of infection [91]. Despite its limitations, the AG129 model has proven to be useful in the testing of a live-attenuated vaccine [134] and in testing the immune response to non-structural proteins in a chimeric vaccine [135]. To improve the model, we have recently developed a new mouse-adapted strain that induces a vascular leak in IFNAR1^−/−^ mice [73]. As these mice lack only the IFN-α/β receptor, they should be more suitable for future vaccine testing.

While the use of NHP models is limited for pathogenesis studies, Rhesus macaques have been shown to develop anti-DENV Abs and viremia kinetics closely related to what is observed in humans [136]. Thus, the read-out for testing vaccines in NHPs is limited to prevention of viremia after challenge and studies on the amount and specificity of the immune response. NHPs have been very useful in predicting the infectivity, replication kinetics and immunogenicity of live-attenuated vaccines that are now in clinical trial and have been used to achieve the development of a balanced tetravalent formulation [137,138,139,140,141,142,143]. 

## 6. Conclusion and Future Directions

The development of a suitable animal model of DENV infection has been hampered by the limited infectivity and replication of DENV in non-human species. Mouse and NHP models are available and each model can be used for different applications. Further manipulation of the virus or the mouse immune system could lead in the future to improved models. For instance, mouse STAT2, in contrast to human STAT2, has recently been shown to restrict DENV infection by resisting degradation mediated by DENV NS5 [144]. Thus, engineering a human STAT2 transgenic mouse could allow the development of an immunocompetent mouse model permissive to DENV infection. New models, such as the use of HLA transgenic mice or the study of human Abs in mice, as well as progress in humanized mice, will allow a better understanding of the human immune response to dengue in an *in vivo* situation. Each model has its advantages and disadvantages; thus, each model should be carefully chosen for different studies, including pathogenesis/immunopathogenesis, development of the immune response, drug testing or vaccine evaluation. 
